# Distinct metabolic profiles associated with autism spectrum disorder versus cancer in individuals with germline *PTEN* mutations

**DOI:** 10.1038/s41525-022-00289-x

**Published:** 2022-03-03

**Authors:** Lamis Yehia, Ying Ni, Tammy Sadler, Thomas W. Frazier, Charis Eng

**Affiliations:** 1 Genomic Medicine Institute, Lerner Research Institute, Cleveland Clinic, Cleveland, OH USA; 2 Center for Immunotherapy and Precision Immuno-Oncology, Lerner Research Institute, Cleveland Clinic, Cleveland, OH USA; 3 Autism Speaks, Cleveland, OH USA; 4 Department of Psychology, John Carroll University, University Heights, OH USA; 5 Taussig Cancer Institute, Cleveland Clinic, Cleveland, OH USA; 6 Department of Genetics and Genome Sciences, Case Western Reserve University School of Medicine, Cleveland, OH USA; 7 Germline High Risk Cancer Focus Group, CASE Comprehensive Cancer Center, Case Western Reserve University, Cleveland, OH USA

**Keywords:** Predictive markers, Cancer genetics, Autism spectrum disorders

## Abstract

PTEN hamartoma tumor syndrome (PHTS), caused by germline *PTEN* mutations, has been associated with organ-specific cancers and autism spectrum disorder (ASD) and/or developmental delay (DD). Predicting precise clinical phenotypes in any one PHTS individual remains impossible. We conducted an untargeted metabolomics study on an age- and sex-matched series of PHTS individuals with ASD/DD, cancer, or both phenotypes. Using agnostic metabolomic-analyses from patient-derived lymphoblastoid cells and their spent media, we found 52 differentially abundant individual metabolites, 69 cell/media metabolite ratios, and 327 pair-wise metabotype (shared metabolic phenotype) ratios clearly distinguishing PHTS individuals based on phenotype. Network analysis based on significant metabolites pointed to hubs converging on PTEN-related insulin, MAPK, AMPK, and mTOR signaling cascades. Internal cross-validation of significant metabolites showed optimal overall accuracy in distinguishing PHTS individuals with ASD/DD versus those with cancer. Such metabolomic markers may enable more accurate risk predictions and prevention in individual PHTS patients at highest risk.

## Introduction

Hereditary cancer predisposition syndromes and neurodevelopmental disorders account for a large subset of individuals in the medical genetics clinic^1–3^. While advances in genomic medicine have enabled the identification of the underlying etiologies for many of these disorders, genotype–phenotype associations are not absolute; it remains challenging to predict the natural history of any one hereditary disorder at the individual patient level versus the population/cohort level^4^. One well-studied model is PTEN hamartoma tumor syndrome (PHTS, MIM 158350), a spectrum of cancer- and neurodevelopmental disorders-related phenotypes caused by germline mutations in the tumor suppressor phosphatase and tensin homolog gene (*PTEN*, MIM 601728)^5,6^. While *PTEN* germline mutations were originally identified in a relatively rare subset of disorders predisposing to breast, thyroid, and other cancers^7^, subsequent studies have shown that *PTEN* germline mutation is amongst the most common causes of autism spectrum disorder (ASD)^8,9^. This *PTEN*-related phenotypic dichotomy poses a challenge for more timely and precise medical management of individuals with germline *PTEN* mutations^6,10^. Deciphering this dichotomy may also have value in identifying the etiology of a subset of ASD/DD.

The extensive phenotypic heterogeneity in PHTS supported the hypothesis that genetic or genomic modifying factors exist. First, earlier studies showed that germline variants in genes encoding three of the four subunits of succinate dehydrogenase or mitochondrial complex II (*SDHB*, *SDHC*, and *SDHD*, collectively referred to as *SDHx*) modify breast cancer risk and thyroid cancer histology in individuals with germline *PTEN* mutations^11,12^. Second, copy number variations were found to be associated with the ASD and/or developmental delay (DD) phenotype versus cancer in patients with germline *PTEN* mutations^13^. These two studies provided proof-of-principle that genomic modifiers may play a role in modulating phenotypic outcomes in PHTS.

Metabolomics, the comprehensive study of small molecules, known as metabolites, in biological systems, has emerged as a promising analytical profiling method for biomarker discovery^14^. Multiple studies have elucidated the association of multiple metabolites and/or metabolic pathways in pathobiological processes, including sporadic cancer and nonsyndromic neurodevelopmental disorders^15–22^. However, the variability among metabolites and metabolic pathways implicated in the same phenotype only reflects the complexity and heterogeneity of such disorders. Recently, metabotyping, a subtyping approach based on shared metabolic phenotypes identified from a set of metabolic biomarkers has shown promise in screening for autism risk in children^17,23^.

Relevant to PHTS and the known role of mitochondrial energetics in this syndrome, we had previously conducted a pilot study using a targeted approach that focused on metabolites within the tricarboxylic acid cycle^16^. This study provided proof-of-principle regarding the role of metabolites as predictive markers of phenotypic outcomes in PHTS. However, because these studies have been limited to a small targeted set of metabolites, the associations we identified thus far represent an incomplete snapshot of metabolites that may influence ASD/DD and/or cancer outcomes in individuals with PHTS.

Integrating metabolomic profiles on top of the genetic predisposition for phenotype prediction is of great interest, especially for inherited disorders like PHTS with seemingly disparate cancer and neurodevelopmental phenotypes. As such, we sought to address the hypothesis that specific metabolites and/or metabolic networks in patients carrying germline *PTEN* mutations are associated with the development of specific clinical phenotypes, here, cancer versus ASD/DD. Thus, we performed a comprehensive untargeted metabolomics approach in a matched series of PHTS individuals.

## Results

### Research participants and study design

**Fig. 1:**
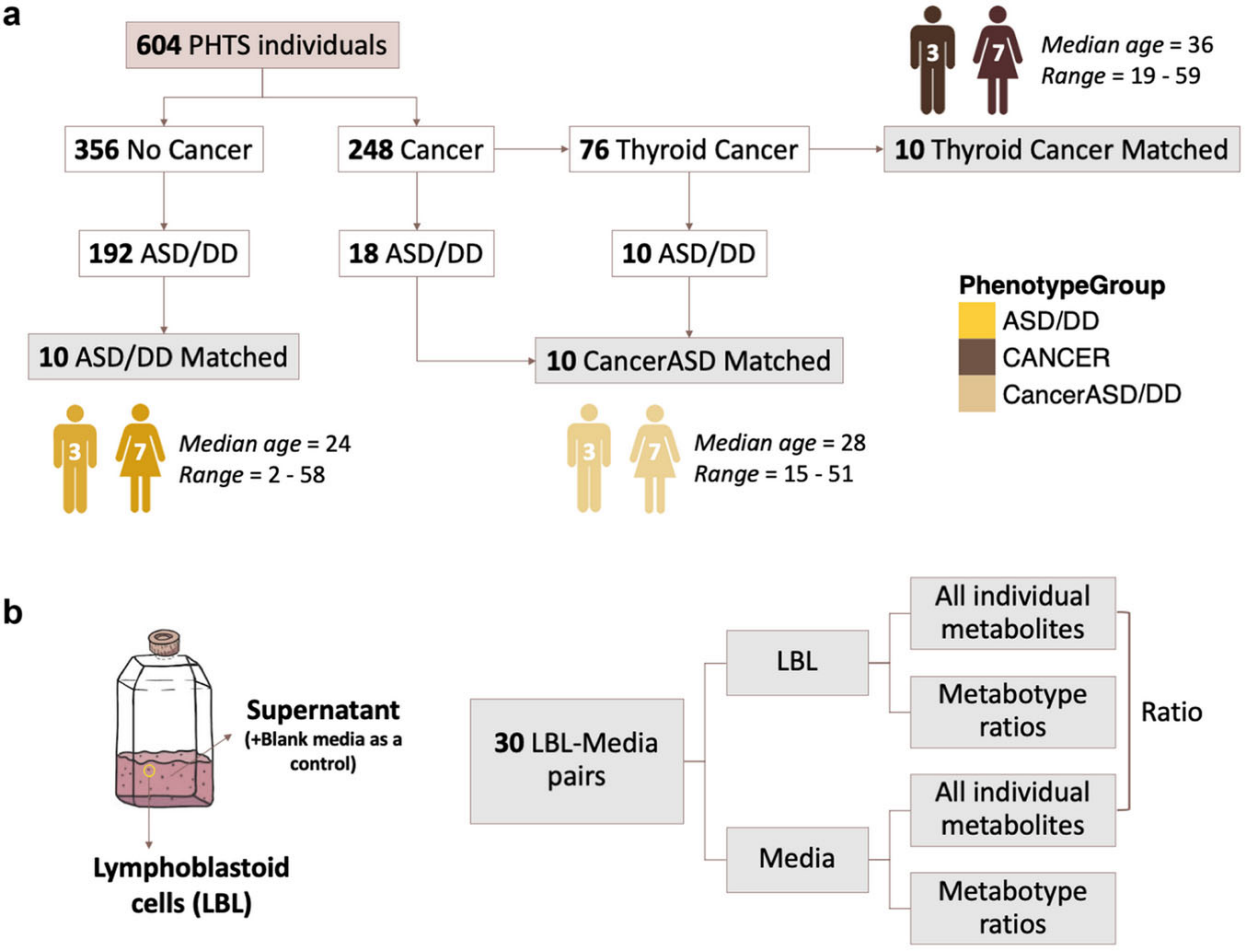
Characteristics of study participants and study design. **a** We selected a series of 30 sex- and age-matched PHTS individuals for untargeted metabolomics measurements. **b** Metabolite sample measurements were obtained from lymphoblastoid cell lines (LBL), paired surrounding growth media for each sample, and blank growth media serving as a baseline negative control. PHTS PTEN hamartoma tumor syndrome, ASD autism spectrum disorder, DD developmental delay.

Six hundred and four individuals diagnosed with PHTS were accrued based on genotypic and phenotypic characteristics and under the approved research protocol 8458-PTEN at the Cleveland Clinic. Of those, 248 (41%) had at least one cancer diagnosis. In this study, we limited the cancer diagnosis to only that of the thyroid since this cancer is the earliest onset cancer type in PHTS affecting both males and females (youngest age at diagnosis starting at 7 years)^24,25^, and importantly, to minimize variability in a deliberately smaller sample size. The earlier age at onset for differentiated thyroid cancer also enables appropriate matching of samples with other young PHTS individuals who have ASD and/or DD. The final matched series consisted of 30 eligible individuals with PHTS (Fig. 1a and Supplementary Table 1). Both females (70%) and males (30%) were represented, and the median age at consent was 28 years (range 2–59). Research participants were divided into three age- and sex-matched groups. The first group consisted of 10 PHTS individuals diagnosed with ASD and/or DD without cancer identified to date, with a median age at consent of 24 years (range 2–58). The second group consisted of 10 PHTS individuals diagnosed with thyroid cancer (median age at consent = 36 years; range = 19–59), with a median age at cancer diagnosis of 25 years (range 17–41). The third group consisted of 10 PHTS individuals (median age at consent = 28 years; range = 15–51) who in addition to ASD/DD, had a cancer diagnosis, with a median age at onset of 18 years (range 7–51). Of note, 7 of the 10 cancer diagnoses consisted of thyroid cancer (Supplementary Table 1). The three phenotype groups were matched with respect to biological sex (*P* = 1, Fisher’s exact test) and ages at consent (*P* = 0.38, effect size = −0.002, Kruskal–Wallis rank sum test).

We generated immortalized lymphoblastoid cell lines (LBLs) from each research participant following standard procedures and then cultured them in vitro for metabolic profiling. Metabolite sample measurements were obtained from LBLs, paired surrounding growth media for each sample, and blank growth media serving as a baseline negative control. We performed analyses examining individual metabolites in each matrix (cells and media), ratio of metabolites shared between the two matrices, and metabotyping analysis focusing on biologically-relevant metabolites (Fig. 1b).

### Identification of single differentially abundant metabolites

Quantified metabolites from LBLs and media consist of amino acids, carbohydrates, cofactors and vitamins, those related to energy metabolism, lipids, nucleotides, peptides, xenobiotics, and partially characterized molecules. Collectively, we detected 645 metabolites from LBLs and 489 metabolites from their matched spent media. As expected, the constitution of measured metabolites differed between the two biological matrices, with enrichment of lipids in the cell compartment compared to the media (Supplementary Fig. 1).

**Fig. 2:**
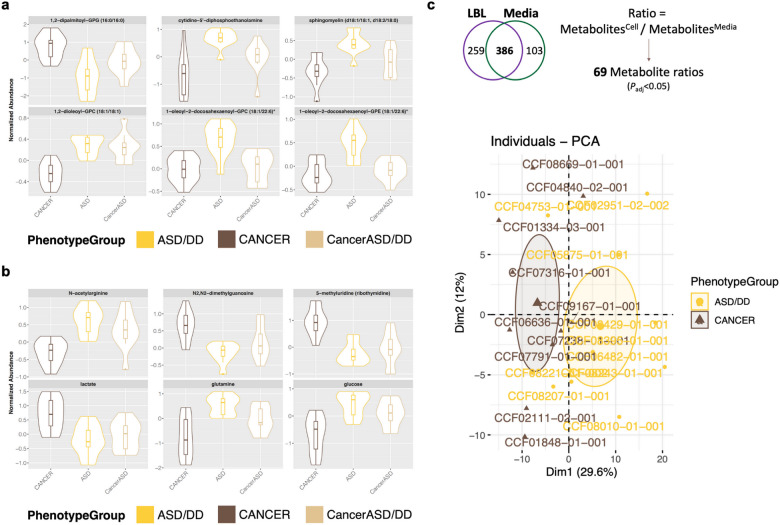
Identification of differentially abundant individual metabolites. **a** Top six differentially abundant metabolites (adjusted *P* < 0.05) amongst cells derived from PHTS individuals belonging to the three phenotype groups. **b** Top six differentially abundant metabolites (adjusted *P* < 0.05) in the surrounding media of cells derived from PHTS individuals. For the violin plots, the center lines represent the median. The lower and upper hinges correspond to the first and third quartiles (the 25th and 75th percentiles). The upper whisker extends from the hinge to the largest value no further than 1.5 × inter-quartile range (IQR) from the hinge. The lower whisker extends from the hinge to the smallest value at most 1.5 × IQR of the hinge. **c** There are 386 metabolites shared in both the cellular and media matrices. Comparisons of the ratios of these metabolites resulted in 69 differentially abundant metabolite ratios (adjusted *P* < 0.05) that successfully separated PHTS individuals according to phenotype group.

We identified multiple differentially abundant metabolites (adjusted *P* < 0.05) amongst samples derived from PHTS individuals with ASD/DD, cancer, and both phenotypes (Supplementary Fig. 2). Using the cellular compartment, we detected multiple differentially abundant metabolites amongst cells derived from PHTS individuals belonging to the three phenotype groups (Fig. 2a and Supplementary Fig. 2). These metabolites generally belong to lipid (89%) and amino acid (11%) classes of molecules (Supplementary Data 1). Similarly, we detected multiple differentially abundant metabolites in the surrounding media of cells derived from PHTS individuals (Fig. 2b and Supplementary Fig. 2). Here, metabolites predominantly belong to amino acids (54%) followed by nucleotides (29%), carbohydrates (8%), cofactors and vitamins (4%), and xenobiotics (4%) (Supplementary Data 1). The principal component analysis (PCA) score plot of metabolic profiles from the media compartment shows clear separation from the blank growth media, serving as a negative control (Supplementary Fig. 3).

Based on our observations interrogating each of the biological matrices (cells and media) on its own (Supplementary Data 2), we then evaluated the ratio of metabolites in cells over spent media, since this method of analysis may uncover biologically-relevant metabolomic alterations that otherwise would not be able to be captured with single metabolite analyses^23,26^. This analysis only applied to metabolites detected in both matrices (Fig. 1b), which led to 386 shared metabolites. There are 69 metabolite cells/media ratios that are significantly different between PHTS individuals with ASD/DD and cancer phenotypes. This analysis revealed a clear separation of PHTS individuals according to phenotype group (Fig. 2c).

### Pathway enrichment analysis

**Fig. 3:**
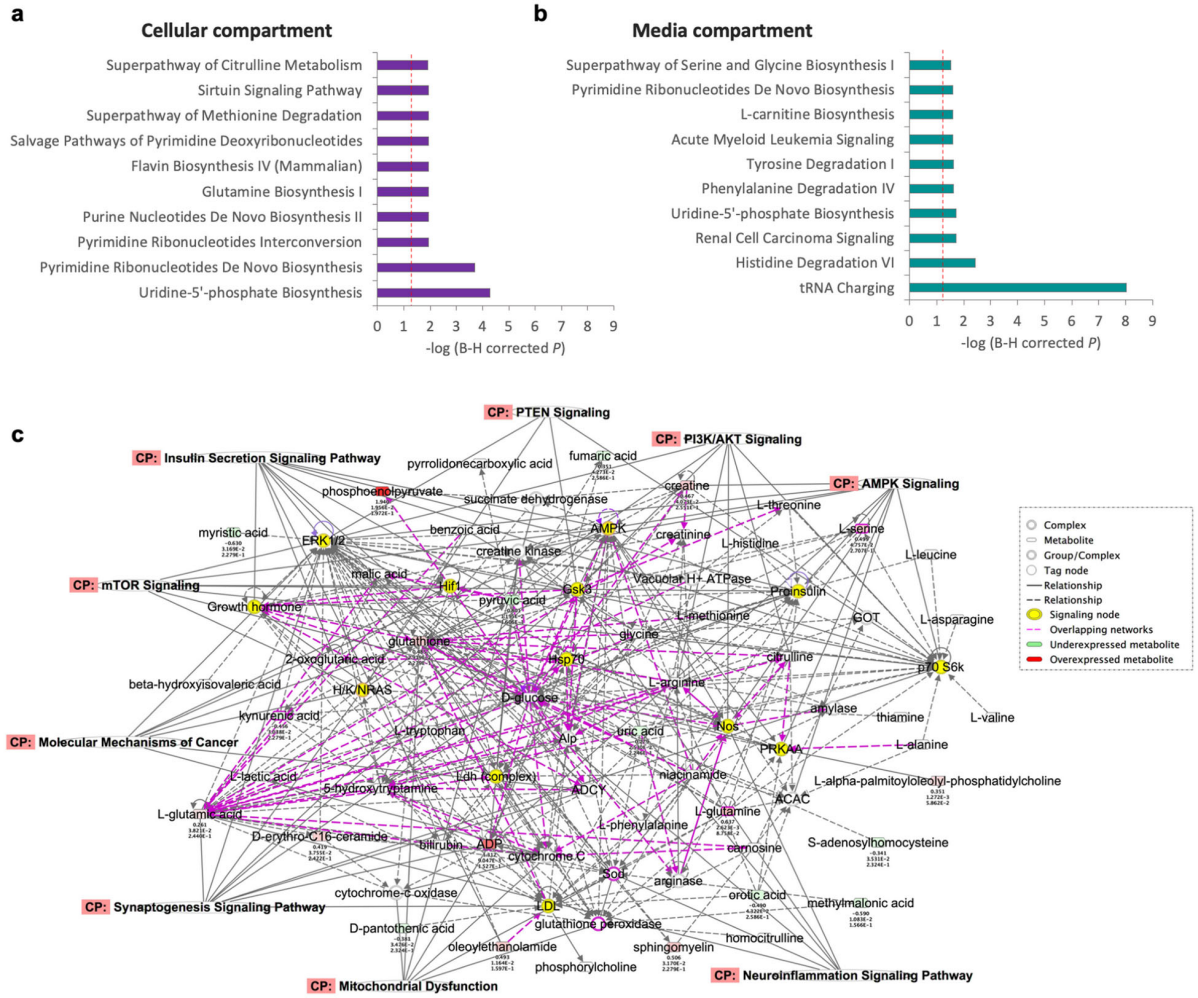
Pathway enrichment analysis and convergence on PTEN-related networks. **a** Top ten most enriched canonical pathways associated with differentially abundant metabolites detected from the cellular compartment. **b** Top ten most enriched canonical pathways associated with differentially abundant metabolites detected from the media compartment. Dashed red lines in (**a**, **b**) indicate the threshold for a significant corrected *P* value < 0.05. **c** Molecular networks integrating all differentially abundant metabolites converge on the PTEN-relevant insulin signaling pathway, as well as ERK1/2, AMPK, and mTOR signaling cascades (*P* ≤ 10^−44^).

To elucidate the biological effects of the observed metabolomic alterations as relevant to cancer and ASD/DD, we performed pathway enrichment analysis. Using the cellular compartment, the top five most enriched canonical pathways comparing ASD/DD versus cancer included glutamine biosynthesis (*P* = 1.2 × 10^−2^), and broadly implicated nucleotide metabolism (*P* ≤ 1.2 × 10^−2^) (Fig. 3a). The top molecular network focusing on diseases and functions implicated cellular compromise, lipid metabolism, and small molecule biochemistry (*P* = 10^−44^). Using the surrounding media, we identify a predominantly amino acid enriched canonical pathway signature, with tRNA charging (*P* = 9.8 × 10^−9^) as the top significant pathway (Fig. 3b). The top molecular network implicated amino acid metabolism, molecular transport, and small molecule biochemistry (*P* = 10^−60^). Finally, the cells/media ratios analysis resulted in only three significantly enriched canonical pathways, including tRNA charging (*P* = 4.3 × 10^−8^), glycine betaine degradation (*P* = 3 × 10^−3^), and glycine biosynthesis (*P* = 7.8 × 10^−3^) (Supplementary Data 3). Similar to the media analysis, the top molecular network included amino acid metabolism, molecular transport, and small molecule biochemistry (*P* = 10^−46^). Intriguingly, the molecular networks from all three analyses converged on the PTEN-relevant insulin signaling pathway, as well as ERK1/2, AMPK, and mTOR signaling cascades (*P* ≤ 10^−44^) (Fig. 3c).

### Metabotype clusters can distinguish PHTS patients with cancer versus those with autism

**Fig. 4:**
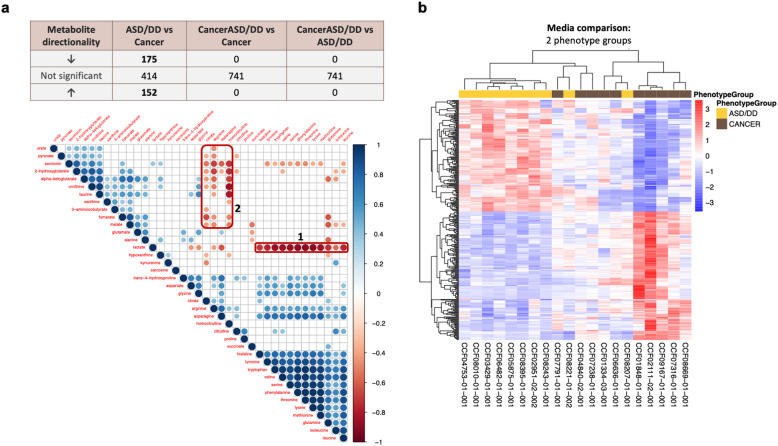
Metabotype clusters can distinguish individuals with cancer versus those with ASD/DD. **a** Differential abundance analysis adjusted for age and sex identified differentially underexpressed and overexpressed metabolite ratio tests in media from samples of PHTS individuals with ASD/DD relative to PHTS individuals with cancer. Correlation plot showing prominent negative correlation blocks (red rectangles). Circles shown are for results significant at *P* < 0.05, with increasing diameter/color corresponding with increasing correlation (circles omitted otherwise). **b** Using the media compartment, unsupervised hierarchical clustering shows that significant differentially abundant metabotypes distinctly separate PHTS individuals with cancer from those who have ASD/DD, with the exception of two patients. ASD autism spectrum disorder, DD developmental delay.

We also implemented a metabotyping approach, subtyping based on shared metabolic phenotypes that has shown promise in risk stratification, including for ASD^17^. Accordingly, guided by our pilot studies and metabolic pathways known to be disrupted in ASD generally^16,17,27,28^, we assessed 43 metabolites associated with amino acid metabolism and mitochondrial energetics in PHTS individuals belonging to the three phenotype groups (Supplementary Table 2). The pair-wise correlation analysis resulted in a total of 703 ratio tests from 38 metabolites detected in the cellular matrix, and 741 ratio tests from 39 metabolites detected in the media matrix. Using the media matrix, we identified 175 differentially underexpressed and 152 overexpressed ratio tests in PHTS individuals with ASD/DD relative to PHTS individuals with cancer (Fig. 4a).

As the negative correlation represents the potential highest pair-wise ratio signal, we observed the most prominent negative correlation blocks for metabolites detected from the media compartment, including lactate versus the amino acid group (block 1) and between arginine, homocitrulline versus serotonin, hydroxyglutarate, ornithine, taurine (block 2) (Fig. 4a). Differential abundance analysis adjusted for age and sex did not identify any significant differentially abundant metabotype clusters in the cellular matrix. As expected, no clear negative correlation block was observed from metabolites detected from cells (Supplementary Fig. 4).

Unsupervised hierarchical clustering shows that significant differentially abundant metabotypes from media distinctly separate PHTS individuals with cancer from those who have ASD/DD, except for two patients (Fig. 4b). Of note, we applied correlation distance measurement and complete clustering methods to delineate group similarities among all individuals included in the heatmap, rather than for classification purposes. The first patient, CCF08221-01-002, is a 2-year-old female with macrocephaly, ASD, global DD, and café-au-lait spots. CCF08207-01-001 is a 46-year-old female with macrocephaly, DD, oral mucosa papilloma, atypical ductal breast hyperplasia, breast fibrocystic disease, intraductal papilloma of breast, noninfiltrating intraductal and lobular carcinoma of the breast, goiter, skin tag, and ovarian cysts.

### Performance of different metabolite predictors

To evaluate the predictive value of metabolite differences we identified to discriminate among phenotypes, we performed internal cross-validation of significant metabolites using the leave one out cross-validation (LOOCV) approach on our relatively small-sized but well-matched samples. This cross-validation indicated optimal sensitivity, specificity, and overall accuracy in distinguishing PHTS individuals with ASD/DD versus those who have cancer using agnostic metabolomic measurements. We compared the classification performance using significant differentially abundant metabolites identified from LBL, media, LBL/media ratio, as well as metabotype ratio, with or without dimension reduction by PCA analysis or removing linearly correlated metabolites. In general, 2-group (Cancer versus ASD) classification performed better than 3-group (Cancer, ASD, and CancerASD) (Supplementary Table 3). In particular, 6 PCA components derived from LBL differentially abundant metabolites showed the highest overall accuracy (0.95, 95% CI: 0.86–0.99, *P* = 3.1 × 10^−14^ compared to No Information Rate [NIR]) distinguishing samples in the ASD/DD group from the cancer group, with a sensitivity of 0.90 and specificity of 1. Metabolite ratios between LBL/media show the second best overall accuracy (0.93, 95% CI: 0.84–0.98, *P* = 4.5 × 10^−13^ compared to NIR) after removing linear correlated ratios, with a sensitivity of 1, and specificity of 0.87 (Table 1).

**Table 1:** Performance metrics.

Matrix	ASD/DD vs. cancer (“positive” class: cancer)	Overall accuracy	Sensitivity	Specificity
Media	AllSigMetabolites (*N* = 23)	0.77	0.73	0.80
	RemoveLinearCorrelated (*N* = 20)	0.77	0.73	0.80
	PCA (*N* = 7)	0.67	0.60	0.73
Cell	AllSigMetabolites (*N* = 8)	0.80	0.80	0.80
	RemoveLinearCorrelated^a^ (*N* = 8)	0.80	0.80	0.80
	PCA (*N* = 6)	**0.95**	**0.90**	**1.00**
Individual metabolite ratio	AllSigMetabolites (*N* = 69)	0.80	0.90	0.70
	RemoveLinearCorrelated (*N* = 20)	**0.93**	**1.00**	**0.87**
	PCA (*N* = 11)	0.87	0.90	0.83
Metabotype ratio	AllSigMetabolites (*N* = 327)	0.62	0.63	0.60
	RemoveLinearCorrelated (*N* = 20)	0.80	0.80	0.80
	PCA (*N* = 9)	0.77	0.80	0.73

*AllSigMetabolites* all significantly abundant metabolites, *PCA* principal component analysis, *ASD* autism spectrum disorder, *DD* developmental delay.

^a^No linear correlated component to be removed. Bolded numbers refer to the comparisons with the highest overall accuracy.

**Fig. 5:**
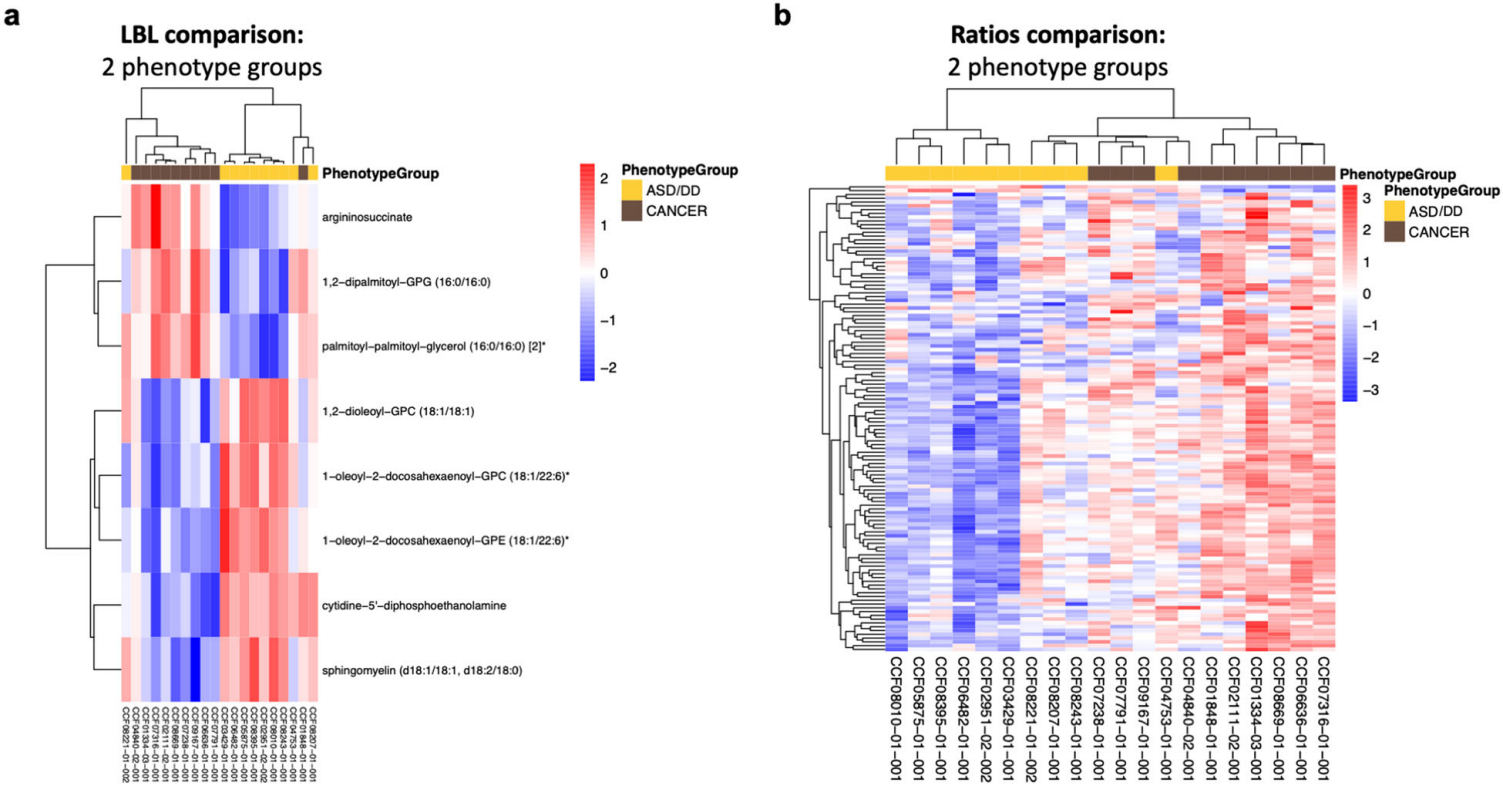
Unsupervised hierarchical clustering can distinguish individuals with cancer versus ASD/DD. **a** Unsupervised hierarchical clustering of differentially abundant metabolites from the cell (LBL) compartment. **b** Unsupervised hierarchical clustering of differentially abundant metabolites resulting from comparisons of ratios of metabolites shared between the cellular and media matrices. ASD autism spectrum disorder, DD developmental delay.

To identify group similarities in metabolomic profiles amongst PHTS individuals with different phenotypes, unsupervised hierarchical clustering of significantly abundant metabolites derived from the LBL component showed that these metabolites distinctly separate PHTS individuals with cancer, with the exception of two patients, from those who have ASD/DD (Fig. 5a). The first patient, CCF08221-01-002, is a 2-year-old female with macrocephaly, ASD, global DD, and café-au-lait spots and was also identified as one of the two misclassified samples in the metabotyping analysis (Fig. 4b). The second patient, CCF01848-01-001, is a 24-year-old female with thyroid cancer, goiter, Hashimoto’s disease, and hemangiomas. PHTS individuals with both cancer and ASD/DD cluster indiscriminately between the PHTS-Cancer and PHTS-ASD/DD groups (Supplementary Fig. 5). The single metabolite ratio analysis distinctly separated PHTS individuals with cancer, except for only one patient, from those who have ASD/DD (Fig. 5b). CCF04753-01-001 is a 42-year-old female with macrocephaly, global DD, goiter, Hashimoto’s disease, breast fibrocystic disease, Lhermitte–Duclos disease (benign hamartomatous overgrowth in the cerebellum), acral keratoses, oral mucosa papillomas, and trichilemmomas.

## Discussion

PHTS represents an excellent model to study phenotypic heterogeneity in the context of a high penetrance Mendelian gene^4,10^. Two of the phenotypes with the most profound consequences in PHTS are cancer and neurodevelopmental disorders, such as ASD^6^. Our efforts have been guided by the principle that identifying markers of specific phenotype risks will enable more precise clinical management and the earliest possible interventions at an individual level. Although it is well established that germline *PTEN* mutations confer a significantly elevated risk of multiple cancer types^25^, it remains unknown whether PHTS children with ASD/DD have identical risks of cancer like PHTS individuals without ASD/DD. This represents a key challenge that would necessitate longitudinal follow-up of PHTS-ASD/DD children into adulthood (the current focus of clinical trial NCT02461446). In parallel, the minority of PHTS-ASD/DD individuals who have already developed cancer (usually in childhood, adolescence, or early adulthood), represent a rare and invaluable sample series to understand cancer etiology compared to neurotypical PHTS individuals. In this study, utilizing a focused series of PHTS individuals with cancer, ASD/DD, or both phenotypes, we attempted to investigate whether an untargeted metabolomics approach may uncover differences in metabolic signatures and signaling networks based on the underlying phenotypes.

We implemented three analytical approaches to show that a subset of metabolites can generate profiles that are distinguishable between PHTS individuals with cancer versus those with ASD/DD. Pathway enrichment and network analysis of significantly abundant metabolites identified a highly interconnected network of metabolites and signaling molecules. Notably, these networks converge on PTEN and associated signaling pathways, including PI3K/AKT, mTOR, insulin, and AMPK. Interestingly, a recent investigation of the urinary proteome in idiopathic autistic and non-autistic children found that differentially abundant proteins converged on processes with known functions in autism, including the PTEN signaling pathway^29^. Another set of metabolites converges on mitochondrial dysfunction and oxidative stress, pathways that have independently been linked to the pathobiology of PHTS and Cowden syndrome associated cancer^11,12^, and to sporadic forms of cancer and “idiopathic” ASD as well^15,17–22,30,31^. Indeed, germline variants in *SDHx* genes encoding mitochondrial complex II can act as genetic modifiers of breast cancer risk and thyroid cancer histology in individuals with PHTS^12^. Ultimately, since cancer and ASD have been thought to share similar underlying molecular etiologies^32,33^, one testable hypothesis is to leverage these differences to preemptively distinguish between these two phenotypes in PHTS.

One of the challenges of studying PHTS is the rarity of the disorder and consequent small sample sizes in many clinical studies. This makes study design crucial. Although this study included only 30 PHTS individuals, we deliberately selected this focused series from >600 PHTS individuals. In addition to a genotypically homogeneous patient sample, we minimize variability through uniform phenotype selection and matching the three phenotype groups by sex and age at consent. Using this strategy to achieve such a powerful experimental design,^4^ despite a limited sample size, facilitated identification of large, biologically-relevant alterations with predictive value at the individual level.

The Children’s Autism Metabolome Project (CAMP, clinical trial NCT02548442) recently found and replicated plasma metabotypes in a series of 499 children with *idiopathic* ASD and 209 typically developing children^17^. Metabotyping is a subtyping approach based on shared metabolic phenotypes identified from a set of metabolic biomarkers^17,23^. CAMP prioritized 39 ASD-focused metabolites associated with amino acid and energy metabolism to identify 34 metabotypes that could detect >50% of autistic participants, which may have clinical value. In this study, we hypothesized that the established ASD metabotypes^17,23^ may have a predictive value in distinguishing PHTS individuals with ASD/DD versus those without ASD/DD, in the research setting. While our data showed acceptable separation of PHTS individuals by ASD/DD versus cancer phenotypes based on metabotype clusters, the overall sensitivity, specificity, and accuracy were lower than the power of individual metabolites from the cellular matrix and the ratio analysis (Table 1). CAMP focused on children 18–48 months of age and quantified metabotypes from plasma^17^. Because we utilized LBLs and their media for metabolite measurements and included PHTS individuals regardless of age, it is plausible that the latter may have metabotype differences involving different metabolite classes than identified by CAMP. Importantly, in our comparisons, all individuals harbored germline *PTEN* mutations, which might obscure signals expected when comparing these PHTS individuals to individuals who are *PTEN* wildtype^16^. These speculations warrant further investigation with more burgeoning data regarding the pathobiology of *PTEN*-related ASD/DD. Importantly, external replication of our internal cross-validation approach will be important for ensuring that small variations in sampling are not significantly impacting model performance in prospective cases.

One intriguing observation is the presence of “misclassified” individuals upon unsupervised hierarchical mapping based on significantly abundant metabolite clusters (Figs. 4b, 5). We speculate that these PHTS-ASD/DD individuals clustered with the PHTS-Cancer group may be at the highest risk of developing cancer during their lifetimes, and longitudinal studies will help test this long-term but important hypothesis. Amongst the three PHTS-ASD/DD individuals clustered with the PHTS-Cancer group using significant abundant metabolites from all analyses (cells, media, and metabotypes), two had a family history of cancer, whereas this information was unknown for the third. However, family history of cancer cannot explain the distinct clustering relative to the other PHTS-ASD/DD individuals forming a uniform cluster. Indeed, of the remaining seven PHTS-ASD/DD individuals, 4 out of 5 with family history information had a positive family history of cancer. Therefore, our findings support our previous observations that family history of cancer alone is not predictive of personal cancer history at the individual level^5^. The alternative, but not mutually exclusive, explanation may be that these cluster “misclassified” ASD/DD individuals are destined to develop malignancies in adulthood. Indeed, one of these PHTS-ASD/DD individuals (CCF08207-01-001) was found to have ductal and lobular carcinoma in situ of the breast, a pre-invasive breast cancer. Conversely, only one PHTS research participant with cancer clustered with the ASD/DD group. This is a 24-year-old female with thyroid cancer, goiter, Hashimoto’s disease, and hemangiomas. To our knowledge, she has not had a formal evaluation for ASD. Hence, we posit that such an approach can help screen for individuals at the highest risk of either phenotype. Unexpectedly, PHTS individuals with both cancer and ASD/DD clustered indiscriminately between the PHTS-Cancer and PHTS-ASD/DD groups. This suggests that this rare subset of PHTS individuals may represent a unique biological subgroup, at least as related to metabolomic profiles.

Overall, this study provides further robust evidence for the roles of relevant metabolites in PHTS-ASD/DD versus PHTS-Cancer etiologies, with the added advantage of having a more homogeneous (monogenic) and well-matched sample series through our study design. Despite research showing the extensive overlap in risk genes (one being *PTEN*) and biological pathways for ASD and for cancer^32,33^, studying such homogeneous patient series will help identify the intricate differences that define the dichotomous phenotype context. The ability to utilize such metabolomic markers will provide a clinical translational framework for stratifying *individual* PHTS patients based on more accurate ASD/DD versus cancer risk predictions, enabling precise risk assessment and early intervention in those at the highest risk.

## Methods

### Research participants and clinical data

A total of 604 individuals clinically diagnosed with *PTEN* hamartoma tumor syndrome were prospectively accrued in accordance with research protocol 8458-*PTEN*, approved by the Cleveland Clinic Institutional Review Board. To address our research question, we prioritized an age- and sex-matched series of 30 individuals with ASD and/or DD without a personal history of invasive cancer(s) (excluding Stage 0 noninfiltrating intraductal and/or lobular carcinoma of the breast), differentiated thyroid cancer, and those with ASD/DD in addition to a cancer diagnosis (majority having thyroid cancer). For each consented research participant, we reviewed medical records, including clinical genetic testing reports, pedigrees, clinical notes associated with cancer genetics and/or genetic-counseling visits, and ASD Diagnostic and Statistical Manual of Mental Disorders (DSM-IV) criteria, where applicable. Written informed consents were obtained from all research participants.

### *PTEN* mutation and deletion analysis

Germline genomic DNA samples from peripheral blood leukocytes were extracted by the Genomic Medicine Biorepository (GMB) of the Cleveland Clinic Genomic Medicine Institute (Cleveland, OH, USA) using standard methods (https://www.lerner.ccf.org/gmi/gmb/). *PTEN* mutation and deletion analysis were performed as previously reported^34^. Mutation analysis was performed with a combination of denaturing gradient gel electrophoresis (DGGE), high-resolution melting curve analysis, and direct Sanger sequencing (ABI 3730xl; Applied Biosystems, Life Technologies) (Supplementary Table 4). Deletion analysis was performed using the multiplex ligation-dependent probe amplification kit (P158; MRC-Holland) according to manufacturer protocol. All patients underwent polymerase chain reaction-based Sanger sequencing of the *PTEN* promoter region. For *PTEN* germline variant positive individuals, pathogenicity predictions are reported according to orthogonal testing in a CLIA-certified facility, ClinVar database classifications, and/or the ClinGen gene-specific criteria for *PTEN* variant curation^35^. Carriers of *PTEN* promoter variants were considered as mutation positive only if the underlying variants have been associated with PHTS or known to affect PTEN function^25,36–38^.

### Cell lines and culture conditions

Immortalized LBLs from peripheral blood samples of individuals with PHTS were generated by the GMB (Cleveland, OH, USA) following standard procedures (https://www.lerner.ccf.org/gmi/gmb/). Cells were subsequently grown in RPMI-1640 supplemented with 20% fetal bovine serum and 1% penicillin/streptomycin and maintained at 37 °C and 5% CO_2_ culture conditions. All cell lines remained anonymized and devoid of any identifiers (only number coded) during the duration of the experiments.

### Sample processing and metabolite measurement template preparation

We seeded the non-adherent LBLs at a density of 10 million cells per T75 flask. At the time of seeding, we transferred 1.5 ml of growth media into three independent aliquots (500 μl each). These media aliquots represent the ‘blank’ metabolite measurements to account for compounds already present in the cell culture media. Cells were allowed to grow overnight and subsequently collected into 50 ml conical tubes. We spun down the cell suspension at 1000 RPM for 5 min in a cooled centrifuge (4 °C). We transferred 1 ml of supernatant from each cell line and saved in fresh and cooled 1.5 ml tubes. These supernatants represent the “secretome” portion of our untargeted metabolomics analysis. We added 1 ml of ice-cold PBS to wash the cell pellet. The cell suspension was then transferred to 1.5 ml tubes, spun down at 1000 RPM for 5 min at 4 °C to remove the PBS wash supernatant. This resulted in ~100 μl of packed cell pellet per sample. All samples (media and cell pellets) were flash frozen using liquid nitrogen and stored at −80 °C.

### Measurement of global untargeted metabolites

Untargeted metabolomics measurements were conducted at Metabolon (Morrisville, NC, USA) using ultrahigh performance liquid chromatography-tandem mass spectrometry (UPLC-MS/MS)^39^. Following receipt by the GMB, samples were inventoried and immediately stored at −80 °C. Following standard procedures to recover metabolites, the resulting extract from each sample was divided into five fractions: two for analysis by two separate reverse phase (RP)/UPLC-MS/MS methods with positive ion mode electrospray ionization (ESI), one for analysis by RP/UPLC-MS/MS with negative ion mode ESI, one for analysis by HILIC/UPLC-MS/MS with negative ion mode ESI, and one sample was reserved for backup. This strategy ensured maximal recovery and coverage of metabolites. All methods utilized a Waters ACQUITY ultra-performance liquid chromatography (UPLC) and a Thermo Scientific Q-Exactive high-resolution/accurate mass spectrometer interfaced with a heated electrospray ionization (HESI-II) source and Orbitrap mass analyzer operated at 35,000 mass resolution. Sample extracts were dried, then reconstituted in solvents compatible to each of the four methods. Each reconstitution solvent contained standards at fixed concentrations to ensure injection and chromatographic consistency. Compounds were identified by comparison to library entries of purified standards or recurrent unknown entities. Metabolon’s library is based on authenticated standards that contains the retention time/index (RI), mass to charge ratio (*m/z)*, and chromatographic data (including MS/MS spectral data) on all molecules present in the library. Additional mass spectral entries have been created for structurally unnamed biochemicals, identified by their recurrent nature (both chromatographic and mass spectral). These compounds have the potential to be identified by future acquisition of a matching purified standard or by classical structural analysis. For the purposes of this study, unknown compounds were excluded from all downstream analyses.

### Data normalization and statistical analyses

For each metabolite, the raw values in the experimental samples are divided by the median of those samples in each instrument batch, giving each batch and thus the metabolite a median of one. The minimum value across all batches in the median scaled data is imputed for the missing values. Metabolites measured from cell pellets are first batch normalized and then divided by the protein concentration, before re-scaling to have median = 1 (divide the new values by the overall median for each metabolite). Afterward, imputation is performed. The batch normalized and imputed data were transformed using the natural log. Final log-transformed and center-scaled metabolite values have a mean of 0 and a standard deviation of 1.

Statistical analyses were conducted with RStudio version 1.4.1717 (https://www.rstudio.com). The linear model was applied to assess differential metabolite expression in the context of a multifactor designed experiment with Limma R package version 3.48.0^40,41^, using age at consent and biological sex as covariates. Furthermore, statistically significant metabolic features were extracted using the criteria of multiple testing corrected *p* value <0.05 and visualized using volcano plots and heatmaps. For pair-wise comparisons, we used two-sided Student’s *t* tests and/or Wilcoxon’s rank sum tests, as indicated. Statistically significant metabolites were then used for networking analysis and pathway enrichment analysis to better understand their biological significance using QIAGEN Ingenuity Pathway Analysis (IPA, QIAGEN, Redwood City, CA).

Internal cross-validation of significant metabolites was performed using LOOCV approach, in which each observation is considered as the validation set and the rest (N − 1) observations are considered as the training set. The model was built on all the data points (metabolite measurements) except one. The left-out data point was then tested with the LogitBoost method using the model built earlier and the test error associated with the prediction was recorded. The process was repeated for all data points and overall prediction error was computed by taking the average of all these test error estimates recorded for each iteration. This cross-validation was conducted using R package Caret (short for Classification And REgression Training, version 6.0.88)^42^, and the performance was evaluated using confusionMatrix function to calculate prediction metrics including sensitivity, specificity, and overall accuracy. Multiple methods were applied to extract the optimal predictors as model input to compare validation performance: (1) all significantly abundant metabolites; (2) significantly abundant metabolites with linear dependencies removed; (3) significantly abundant metabolites transformed using PCA to a smaller sub-space where the new PCA variables are uncorrelated with one another.

### Reporting summary

Further information on research design is available in the Nature Research Reporting Summary linked to this article.

## Supplementary information

Supplementary material

Dataset 1

Dataset 2

Dataset 3

Dataset 4

Dataset 5

Reporting Summary

## Data Availability

Raw metabolomics data and analyses related to this study are included in the figures, table, and Supplementary Data 1–5.

## Code availability

All software versions are indicated within the Methods. All custom scripts related to this study are available from the corresponding author upon reasonable request.
