# Supplementary material for: Distinct metabolic profiles associated with autism spectrum disorder versus cancer in individuals with germline PTEN mutations
Source: NPJ Genom Med. 2022 Mar 3;7:16. doi: 10.1038/s41525-022-00289-x (PMC8894426; doi:10.1038/s41525-022-00289-x)
Supplement: Supplementary file 1 — Supplementary material [file 41525_2022_289_MOESM1_ESM.pdf]

Supplementary Information

Distinct metabolic profiles associated with autism spectrum disorder versus cancer in individuals with germline *PTEN* mutations

Lamis Yehia, Ying Ni, Tammy Sadler, Thomas W. Frazier, Charis Eng

Contents

Supplementary Figures: 5

Supplementary Tables: 4

Supplementary Data: 5 (separate Excel datasets)

Figures

Supplementary Figure 1: Abundance of metabolites from lymphoblastoid cells and spent media

Supplementary Figure 2: Identification of differentially abundant metabolites and principal component analysis (PCA) of metabolic profiles from the cell/media metabolite ratios

Supplementary Figure 3: Principal component analysis score plot of metabolic profiles from media

Supplementary Figure 4: Metabotype cluster analysis in the cellular matrix

Supplementary Figure 5: PHTS individuals with both cancer and ASD/DD cluster indiscriminately between the PHTS-Cancer and PHTS-ASD/DD groups

Tables

Supplementary Table 1: Demographic and clinical characteristics of thirty matched PHTS individuals

Supplementary Table 2: Metabolites analysed through the metabotyping analysis

Supplementary Table 3: Performance metrics of differentially abundant metabolites

Supplementary Table 4: *PTEN* primers used for high-resolution melting analysis and Sanger sequencing

Supplementary Figure 1: Abundance of metabolites from lymphoblastoid cells (LBL) and spent media

A.

	Cells (LBL)	Media
Amino Acids	163	169
Carbohydrates	39	24
Cofactors and Vitamins	32	23
Energy	9	12
Lipids	276	146
Nucleotides	67	45
Partially Characterized Molecules	1	1
Peptides	29	19
Xenobiotics	29	50
**Total**	**645**	**489**

B.

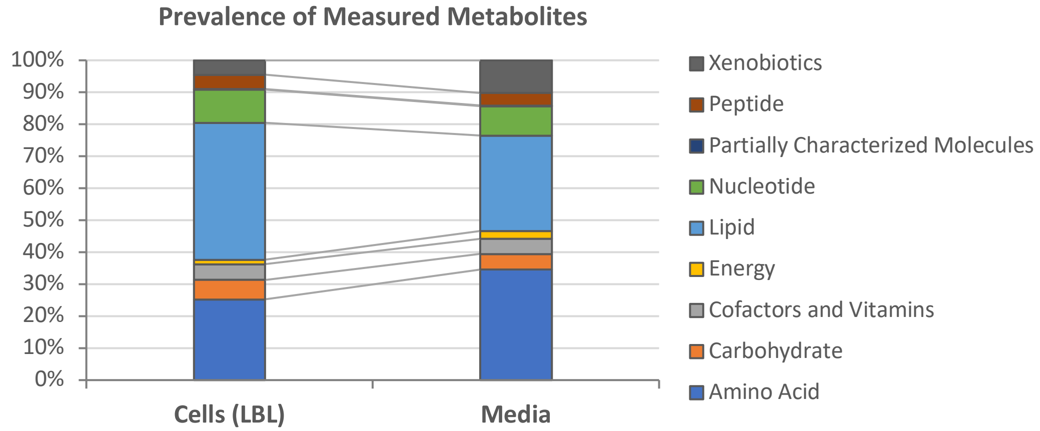

C.

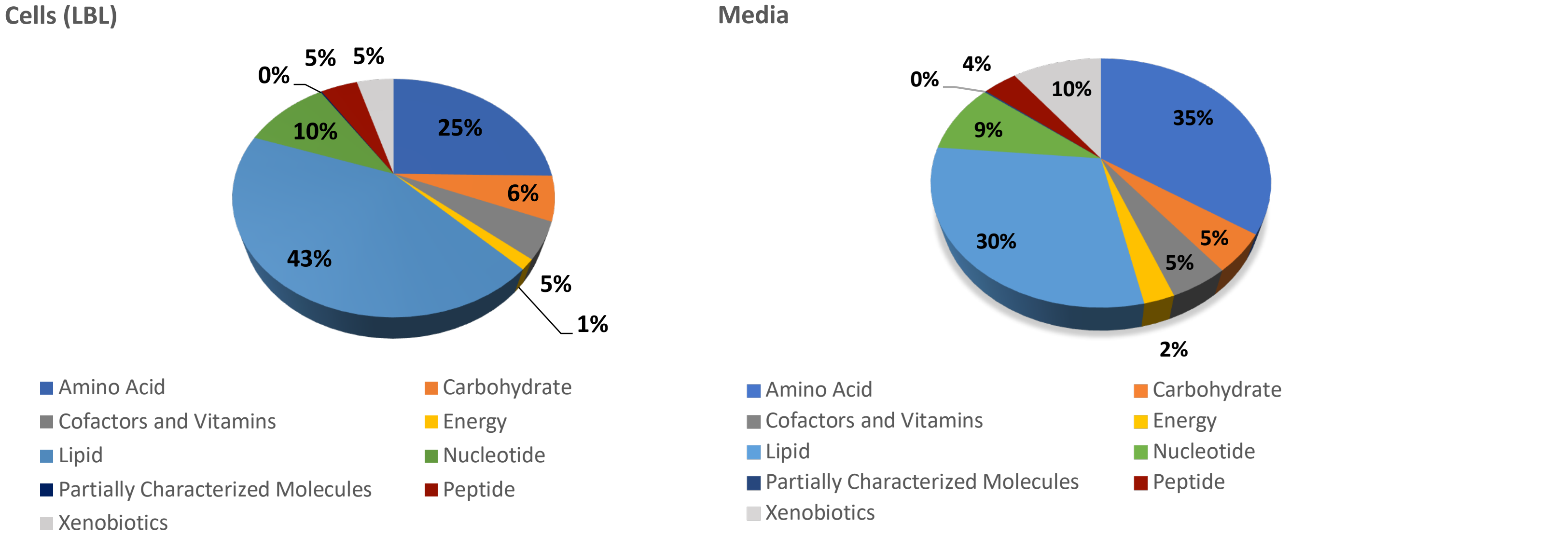

A. Number of metabolites identified for each of the cellular and media compartments. **B-C.** Prevalence of the measured metabolites represented as a bar graph or within pie charts. *Abbreviations*: LBL, lymphoblastoid cell line

Supplementary Figure 2: Identification of differentially abundant metabolites and principal component analysis (PCA) of metabolic profiles from the cell/media metabolite ratios

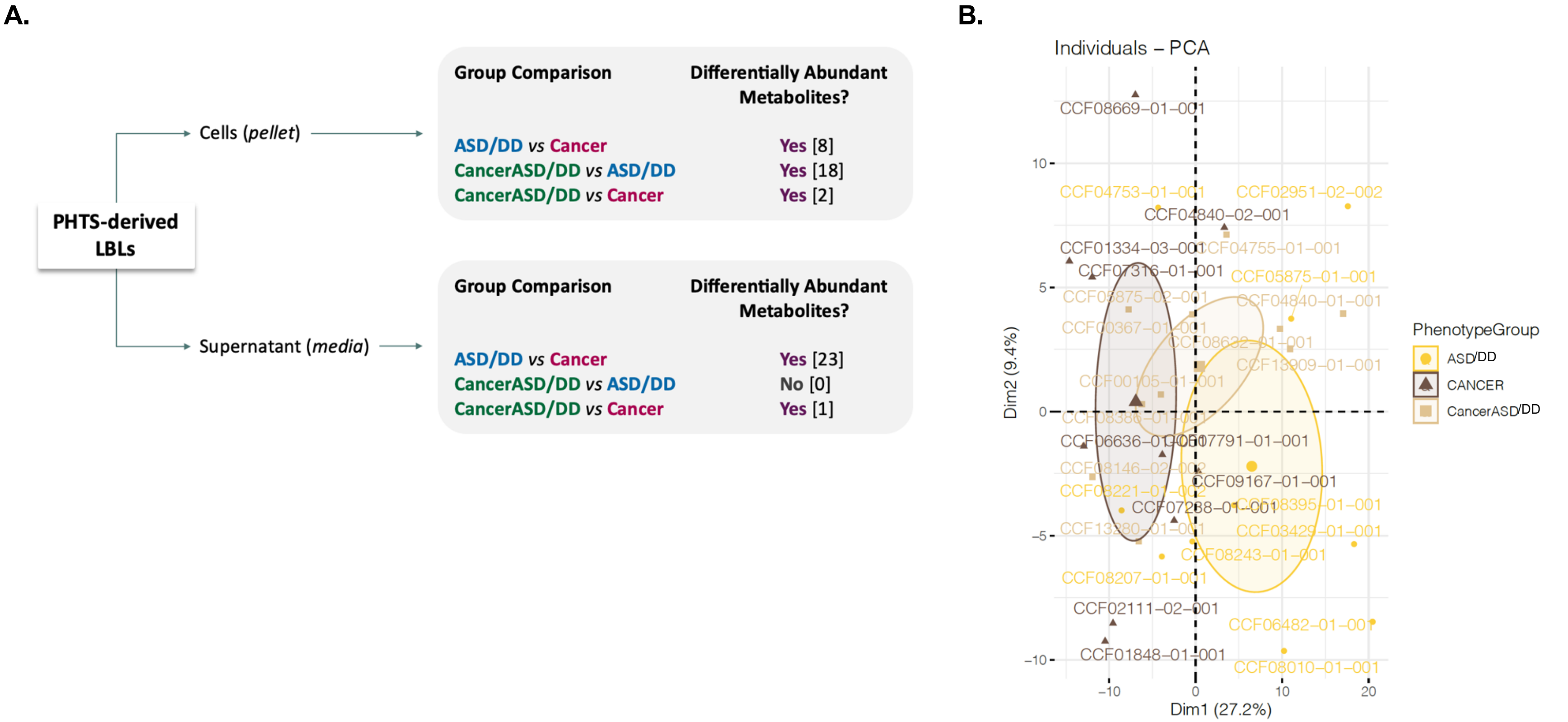

A. Differential abundance analysis adjusted for age and sex identified differentially abundant metabolites (adjusted *P*<0.05; the number of significant metabolites depicted in square brackets for each comparison). **B.** Principal component analysis (PCA) score plot of metabolic profiles from the cell/media metabolite ratios shows clear separation between PHTS-ASD/DD and PHTS-Cancer groups. PHTS individuals with both cancer and ASD/DD clustered indiscriminately between the PHTS-Cancer and PHTS-ASD/DD groups. *Abbreviations*: LBLs, lymphoblastoid cell lines; ASD, autism spectrum disorder; DD, developmental delay.

Supplementary Figure 3: Principal component analysis score plot of metabolic profiles from media

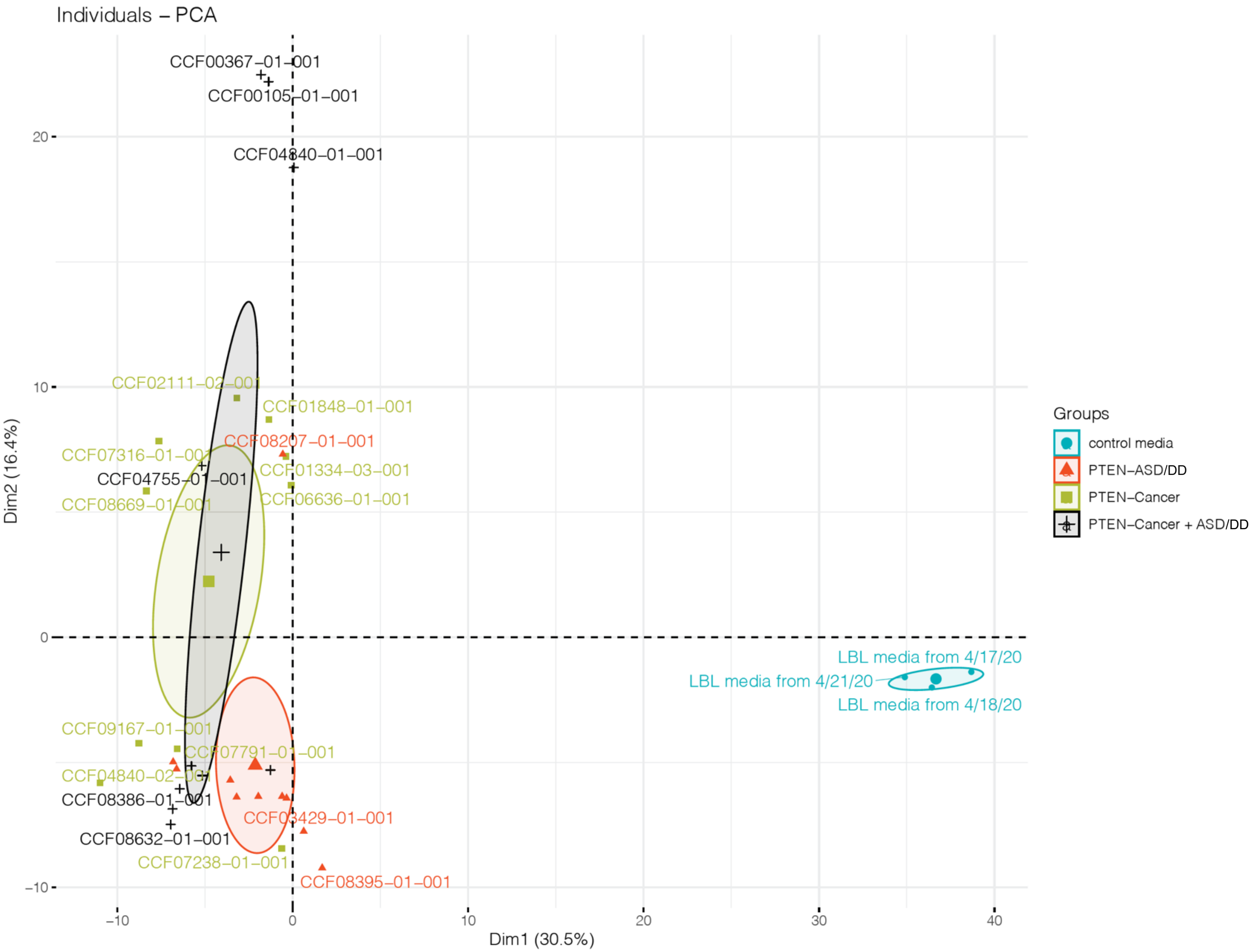

Principal component analysis (PCA) score plot of metabolic profiles from the media compartment (black, red, and green clusters) shows clear separation from the blank growth media (blue cluster), serving as a negative control. *Abbreviations*: ASD, autism spectrum disorder; DD, developmental delay.

Supplementary Figure 4: Metabotype cluster analysis in the cellular matrix

A.

Metabolite Directionality	ASD/DD vs Cancer	CancerASD/DD vs Cancer	CancerASD/DD vs ASD/DD
↓	0	0	0
Not significant	703	703	703
↑	0	0	0

B.

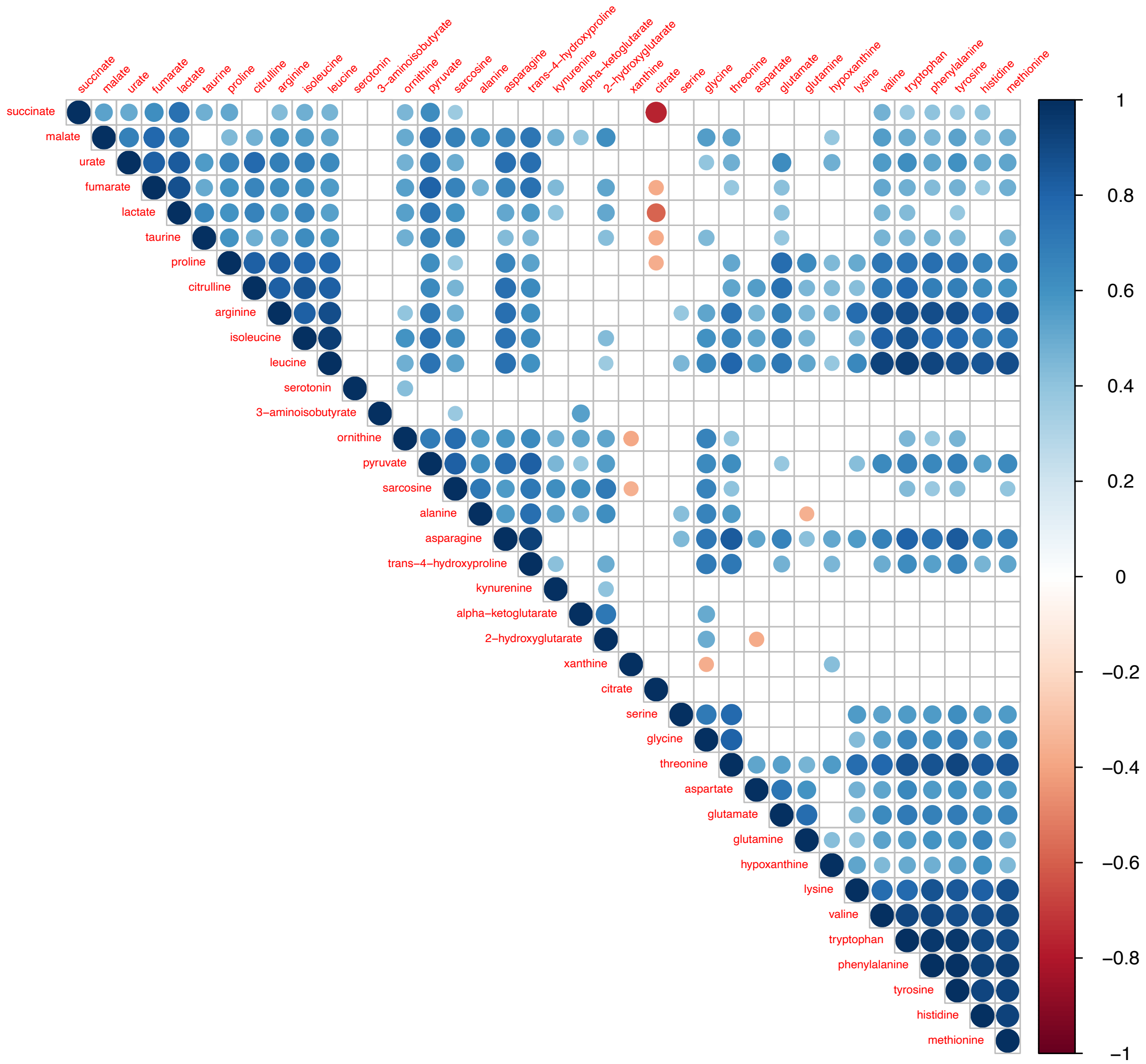

A. Differential abundance analysis adjusted for age and sex did not identify any significant differentially abundant metabotype clusters in the cellular matrix. **B.** Correlation plot showing no clear negative correlation blocks (clusters of red circles) from metabolites detected from the cellular compartment. *Abbreviations*: ASD, autism spectrum disorder; DD, developmental delay.

Supplementary Figure 5: PHTS individuals with both cancer and ASD/DD cluster indiscriminately between the PHTS-Cancer and PHTS-ASD/DD groups

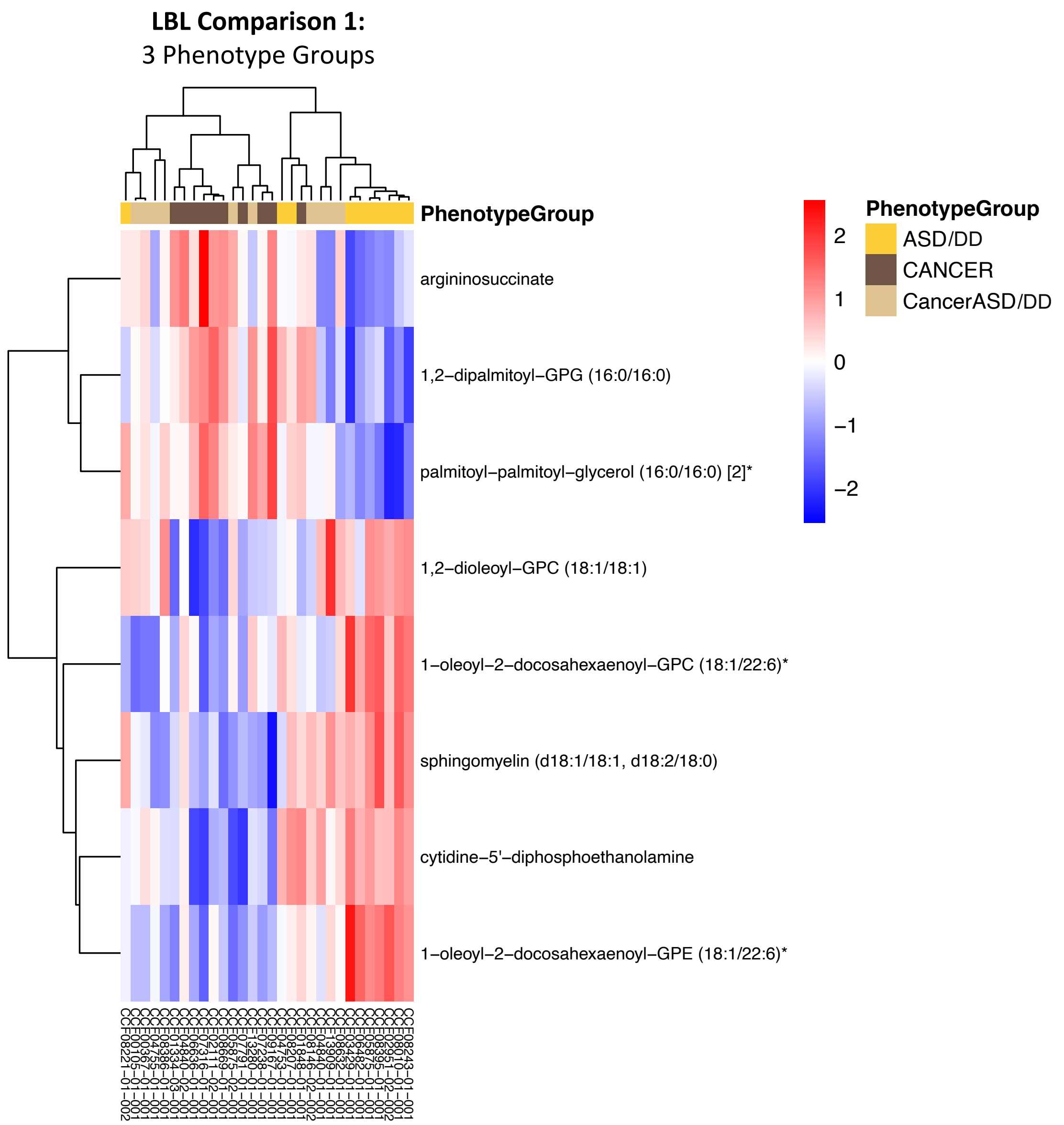

Unsupervised hierarchical clustering of cell-derived single differentially abundant metabolites amongst the three PHTS phenotype groups (ASD/DD, Cancer, and CancerASD). *Abbreviations*: LBL, lymphoblastoid cell lines; ASD, autism spectrum disorder; DD, developmental delay.

Supplementary Table 1: Demographic and clinical characteristics of thirty matched PHTS individuals

Participant ID	Phenotype	Sex	Age at consent	Age at diagnosis of cancer	Germline *PTEN* variants
CCF00105-01-001	ASD/DD+Cancer-Thyroid	M	26	7	p.Lys164Argfs*3
CCF00367-01-001	ASD/DD+Cancer-Thyroid	F	29	14	p.Glu291Lysfs*16
CCF04755-01-001	ASD/DD+Cancer-Thyroid	F	21	17	p.Ser170Ile
CCF04840-01-001	ASD/DD+Cancer-Thyroid	F	35	9	p.Lys254Thr
CCF05875-02-001	ASD/DD+Cancer-Thyroid	M	51	51	p.Arg335*
CCF08146-02-002	ASD/DD+Cancer-Kidney	M	47	21	p.Ser113fs
CCF08386-01-001	ASD/DD+Cancer-Thyroid	F	27	18	p.Phe258fs
CCF08632-01-001	ASD/DD+Cancer-GU	F	20	20	p.Tyr155His
CCF13280-01-001	ASD/DD+Cancer-Thyroid	F	15	14	p.Gly129Val
CCF13909-01-001	ASD/DD+Cancer-GI/GU	F	49	45	p.Arg335*
CCF02951-02-002	ASD/DD	F	58	-	p.Arg14del
CCF03429-01-001	ASD/DD	F	19	-	p.Thr26Pro
CCF04753-01-001	ASD/DD	F	42	-	p.Arg130*
CCF05875-01-001	ASD/DD	M	23	-	p.Arg335*
CCF06482-01-001	ASD/DD	F	14	-	p.Asp252Val
CCF08010-01-001	ASD/DD	M	24	-	p.Cys211fs
CCF08207-01-001	ASD/DD	F	46	-	p.Cys136Arg
CCF08221-01-002	ASD/DD	F	2	-	p.Arg173Cys
CCF08243-01-001	ASD/DD	M	27	-	c.802-2A>G
CCF08395-01-001	ASD/DD	F	5	-	p.Met35Val
CCF01334-03-001	Cancer-Thyroid	F	19	17	c.210-1G>A
CCF01848-01-001	Cancer-Thyroid	F	24	24	p.Arg130*
CCF02111-02-001	Cancer-Thyroid	M	43	41	c.80-?_164+?del (E2 del)
CCF04840-02-001	Cancer-Thyroid	F	59	27	p.Lys254Thr
CCF06636-01-001	Cancer-Thyroid	F	19	19	p.Leu70Pro
CCF07238-01-001	Cancer-Thyroid	F	40	21	p.Arg130Leu
CCF07316-01-001	Cancer-Thyroid	M	32	26	p.Arg130*
CCF07791-01-001	Cancer-Thyroid	F	40	30	p.Tyr88*
CCF08669-01-001	Cancer-Thyroid	M	45	34	c.634+5G>C, p.Gly165Ilefs*9
CCF09167-01-001	Cancer-Thyroid	F	32	24	c.-1170C>T

Abbreviations: ASD/DD, autism spectrum disorder and/or developmental delay; GU, genitourinary; GI, gastrointestinal; M, male; F, female; E2 del, exon 2 deletion.

Supplementary Table 2: Metabolites analysed through the metabotyping analysis

No.	Compound	Class	Pathway
1	hydroxyproline	Amino acid	Urea cycle; Arginine and Proline Metabolism
2	alpha-ketoglutarate	Energy	Tricarboxylic acid (TCA) cycle
3	alanine	Amino acid	Alanine and Aspartate Metabolism
4	arginine	Amino acid	Urea cycle; Arginine and Proline Metabolism
5	asparagine	Amino acid	Alanine and Aspartate Metabolism
6	aspartate	Amino acid	Alanine and Aspartate Metabolism
7	3-aminoisobutyrate	Nucleotide	Pyrimidine Metabolism, Thymine containing
8	citrulline	Amino acid	Urea cycle; Arginine and Proline Metabolism
9	ethanolamine	Lipid	Phospholipid Metabolism
10	2-aminobutyrate	Peptide	Gamma-glutamyl Amino Acid
11	glutamate	Amino acid	Glutamate metabolism
12	glutamine	Amino acid	Glutamate metabolism
13	glycine	Amino acid	Glycine, Serine and Threonine Metabolism
14	histidine	Amino acid	Histidine metabolism
15	homocitrulline	Amino acid	Urea cycle; Arginine and Proline Metabolism
16	homoserine	Amino acid	Glycine, Serine and Threonine Metabolism
17	hypoxanthine	Nucleotide	Purine Metabolism, (Hypo)Xanthine/Inosine containing
18	isoleucine	Amino acid	Leucine, Isoleucine and Valine Metabolism
19	kynurenine	Amino acid	Tryptophan Metabolism
20	lactate	Carbohydrate	Glycolysis, Gluconeogenesis, and Pyruvate Metabolism
21	leucine	Amino acid	Leucine, Isoleucine and Valine Metabolism
22	lysine	Amino acid	Lysine metabolism
23	methionine	Amino acid	Methionine, Cysteine, SAM and Taurine Metabolism
24	ornithine	Amino acid	Urea cycle; Arginine and Proline Metabolism
25	phenylalanine	Amino acid	Phenylalanine metabolism
26	proline	Amino acid	Urea cycle; Arginine and Proline Metabolism
27	pyruvate	Carbohydrate	Glycolysis, Gluconeogenesis, and Pyruvate Metabolism
28	sarcosine	Amino acid	Glycine, Serine and Threonine Metabolism
29	serine	Amino acid	Glycine, Serine and Threonine Metabolism
30	serotonin	Amino acid	Tryptophan Metabolism
31	succinate	Energy	Tricarboxylic acid (TCA) cycle
32	taurine	Amino acid	Methionine, Cysteine, SAM and Taurine Metabolism
33	threonine	Amino acid	Glycine, Serine and Threonine Metabolism
34	tryptophan	Amino acid	Tryptophan Metabolism
35	tyrosine	Amino acid	Tyrosine Metabolism
36	urate	Nucleotide	Purine Metabolism, (Hypo)Xanthine/Inosine containing
37	valine	Amino acid	Leucine, Isoleucine and Valine Metabolism
38	xanthine	Nucleotide	Purine Metabolism, (Hypo)Xanthine/Inosine containing
39	citrate	Energy	Tricarboxylic acid (TCA) cycle
40	isocitrate	Energy	Tricarboxylic acid (TCA) cycle
41	fumarate	Energy	Tricarboxylic acid (TCA) cycle
42	malate	Energy	Tricarboxylic acid (TCA) cycle
43	2-hydroxyglutarate	Lipid	Fatty Acid, Dicarboxylate

Supplementary Table 3: Performance metrics of differentially abundant metabolites

			Sensitivity			Specificity		
Individual Metabolites		***Total Accuracy***	***Cancer***	***ASD/DD***	***CancerASD/DD***	***Cancer***	***ASD/DD***	***CancerASD/DD***
**Media**	AllSigMetabolites (N=121)	0.60	0.62	0.67	0.50	0.85	0.86	0.68
	RemoveLinearCorrelated (N=30)	0.52	0.48	0.62	0.46	0.70	0.89	0.70
	PCA (N=21)	0.40	0.28	0.59	0.30	0.62	0.67	0.81
		Total Accuracy	***Cancer***	***ASD/DD***	***CancerASD/DD***	***Cancer***	***ASD/DD***	***CancerASD/DD***
**Cell**	AllSigMetabolites (N=216)	0.70	0.67	0.70	0.73	0.88	0.83	0.84
	RemoveLinearCorrelated (N=30)	0.68	0.46	0.79	0.81	0.94	0.85	0.73
	PCA (N=22)	0.71	0.70	0.68	0.75	0.94	0.76	0.88
		Total Accuracy	***Cancer***	***ASD/DD***	***CancerASD/DD***	***Cancer***	***ASD/DD***	***CancerASD/DD***
**Ratio**	AllSigMetabolites (N=162)	0.59	0.63	0.52	0.61	0.82	0.89	0.67
	RemoveLinearCorrelated (N=30)	0.59	0.81	0.52	0.44	0.80	0.79	0.78
	PCA (N=21)	0.46	0.48	0.38	0.50	0.80	0.72	0.67
			**Sensitivity**			**Specificity**		
Metabotype Ratio		***Total Accuracy***	***Cancer***	***ASD/DD***	***CancerASD/DD***	***Cancer***	***ASD/DD***	***CancerASD/DD***
**Media**	AllSigMetabolites (N=327)	0.59	0.75	0.70	0.31	0.81	0.84	0.72
	RemoveLinearCorrelated (N=29)	0.29	0.43	0.36	0.08	0.70	0.68	0.57
	PCA (N=11)	0.51	0.48	0.62	0.43	0.83	0.77	0.65

Abbreviations: AllSigMetabolites, all significantly abundant metabolites; PCA, principal component analysis; ASD, autism spectrum disorder; DD, developmental delay.

Supplementary Table 4: *PTEN* primers used for high-resolution melting analysis and Sanger sequencing

Name	Sequence 5’ -> 3’	Annealing Temp (°C)
PTEN LS E1F<br>PTEN LS E1R	GCAGCTTCTGCCATCTC<br>GCATCCGTCTACTCCCAC	60
PTEN LS E2F<br>PTEN LS E2R	AGTATTCTTTTAGTTTGATTGCTGC<br>CTAAATGAAAACACAACATGAATATAAACA	60
PTEN LS E3F<br>PTEN LS E3R	ATGTTAGCTCATTTTTGTTAATGGTG<br>CAAGCAGATAACTTTCACTTAATAGTTG	60
PTEN LS E4F<br>PTEN LS E4R	TTTTTTCTTCCTAAGTGCAAAAGATAAC<br>CAGTAAGATACAGTCTATCGGGT	60
PTEN LS E5F<br>PTEN LS E5R<br>PTEN LS E5SeqF	ACCTACTTGTTAATTAAAAATTCAAGAGTT<br>ATCCAGGAAGAGGAAAGGAAA<br>TGCAACATTTCTAAAGTTACCTACTTG	60<br><br>For sequencing
PTEN LS E6F<br>PTEN LS E6R	CCCAGTTACCATAGCAATTTAGTGA<br>TAGATATGGTTAAGAAAACTGTTCCAATAC	60
PTEN LS E7F<br>PTEN LS E7R	CAGTTTGACAGTTAAAGGCATTTC<br>AATATAGCTTTTAATCTGTCCTTATTTTGG	61
PTEN LS E8.1F<br>PTEN LS E8.1R	TTTGTTGACTTTTTGCAAATGTTTAACATA<br>ATTTCTTGATCACATAGACTTCCA	61
PTEN LS E8.2F<br>PTEN LS E8.2R	GTAAATACATTCTTCATACCAGGACC<br>GCTGTACTCCTAGAATTAAACACAC	61
PTEN LS E9F<br>PTEN LS E9R<br>PTEN LS E9SeqF<br>PTEN LS E9SeqR	AAGATGAGTCATATTTGTGGGTT<br>TTTCAGTTTATTCAAGTTTATTTTCATGG<br>AGATGAGTCATATTTGTGGGTTTT<br>AAAGGTCCATTTTCAGTTTATTCAA	61<br><br>For sequencing<br>For sequencing
PTEN LS SNP E8.2F<br>PTEN LS SNP E8.2R<br>PTEN LS SNP E8.2 Probe<br>PTEN-Promoter1F<br>PTEN-Promoter1R	GCAAATAAAGACAAAGCCAACCGA<br>AGCTGTACTCCTAGAATTAAACACACATC<br>CATACAAGTCACCAACCCCCAC-block<br>GCGTGGTCACCTGGTCCTTT<br>GCTGCTCACAGGCGCTGA	55<br>For Intronic 8 SNP<br><br>62<br>62
