# Supplementary material for: Distinct metabolic profiles associated with autism spectrum disorder versus cancer in individuals with germline PTEN mutations
Source: NPJ Genom Med. 2022 Mar 3;7:16. doi: 10.1038/s41525-022-00289-x (PMC8894426; doi:10.1038/s41525-022-00289-x)
Supplement: Supplementary file 7 — Reporting Summary [file 41525_2022_289_MOESM7_ESM.pdf]

nature portfolio

Corresponding author(s): Charis Eng, MD, PhD

Last updated by author(s): Oct 14, 2021

Reporting Summary

Nature Portfolio wishes to improve the reproducibility of the work that we publish. This form provides structure for consistency and transparency in reporting. For further information on Nature Portfolio policies, see our Editorial Policies and the Editorial Policy Checklist.

Statistics

For all statistical analyses, confirm that the following items are present in the figure legend, table legend, main text, or Methods section.

n/a	Confirmed	
☐	☒	The exact sample size (*n*) for each experimental group/condition, given as a discrete number and unit of measurement
☐	☒	A statement on whether measurements were taken from distinct samples or whether the same sample was measured repeatedly
☐	☒	The statistical test(s) used AND whether they are one- or two-sided<br>*Only common tests should be described solely by name; describe more complex techniques in the Methods section.*
☐	☒	A description of all covariates tested
☐	☒	A description of any assumptions or corrections, such as tests of normality and adjustment for multiple comparisons
☐	☒	A full description of the statistical parameters including central tendency (e.g. means) or other basic estimates (e.g. regression coefficient) AND variation (e.g. standard deviation) or associated estimates of uncertainty (e.g. confidence intervals)
☐	☒	For null hypothesis testing, the test statistic (e.g. *F*, *t*, *r*) with confidence intervals, effect sizes, degrees of freedom and *P* value noted<br>*Give P values as exact values whenever suitable.*
☐	☒	For Bayesian analysis, information on the choice of priors and Markov chain Monte Carlo settings
☐	☒	For hierarchical and complex designs, identification of the appropriate level for tests and full reporting of outcomes
☐	☒	Estimates of effect sizes (e.g. Cohen's *d*, Pearson's *r*), indicating how they were calculated

Our web collection on statistics for biologists contains articles on many of the points above.

Software and code

Policy information about availability of computer code

Data collection	For metabolite measurements, the Metabolon informatics system consists of four major components: the Laboratory Information Management System (LIMS), the data extraction and peak-identification software, data processing tools for QC and compound identification, and a collection of information interpretation and visualization tools for use by data analysts. Hardware and software foundations for these informatics components are the LAN backbone, and a database server running Oracle 10.2.0.1 Enterprise Edition.
Data analysis	RStudio version 1.4.1717<br>Limma R package version 3.48.0<br>Caret R package version 6.0.88<br>QIAGEN Ingenuity Pathway Analysis Summer 2021 release

For manuscripts utilizing custom algorithms or software that are central to the research but not yet described in published literature, software must be made available to editors and reviewers. We strongly encourage code deposition in a community repository (e.g. GitHub). See the Nature Portfolio guidelines for submitting code & software for further information.

March 2021

Data

Policy information about availability of data

All manuscripts must include a data availability statement. This statement should provide the following information, where applicable:

- Accession codes, unique identifiers, or web links for publicly available datasets
- A description of any restrictions on data availability
- For clinical datasets or third party data, please ensure that the statement adheres to our policy

Any raw data or analyses related to this study are available from the corresponding author upon reasonable request. All metabolomic data are available in Supplementary Data 1.

Field-specific reporting

Please select the one below that is the best fit for your research. If you are not sure, read the appropriate sections before making your selection.

☒ Life sciences ☐ Behavioural & social sciences ☐ Ecological, evolutionary & environmental sciences

For a reference copy of the document with all sections, see nature.com/documents/nr-reporting-summary-flat.pdf

Life sciences study design

All studies must disclose on these points even when the disclosure is negative.

Sample size	No sample size calculation was performed.
Data exclusions	Unless otherwise specified, only metabolites with adjusted p-values < 0.05 were included in downstream analyses.
Replication	We performed internal cross-validation of significant metabolites using Leave One Out Cross-Validation approach, in which each observation is considered as the validation set and the rest (N-1) observations are considered as the training set.
Randomization	PHTS individuals were divided into 3 groups based on phenotype: (1) autism spectrum disorder (ASD) and/or developmental delay (DD), (2) cancer, and (3) those with ASD/DD in addition to a cancer diagnosis. Age and biological sex were used as covariates.
Blinding	Metabolite measurements were collected in a blinded manner using coded samples.

Reporting for specific materials, systems and methods

We require information from authors about some types of materials, experimental systems and methods used in many studies. Here, indicate whether each material, system or method listed is relevant to your study. If you are not sure if a list item applies to your research, read the appropriate section before selecting a response.

Materials & experimental systems

n/a	Involved in the study	
☒	☐	Antibodies
☒	☐	Eukaryotic cell lines
☒	☐	Palaeontology and archaeology
☒	☐	Animals and other organisms
☐	☒	Human research participants
☒	☐	Clinical data
☒	☐	Dual use research of concern

Methods

n/a	Involved in the study	
☒	☐	ChIP-seq
☒	☐	Flow cytometry
☒	☐	MRI-based neuroimaging

Human research participants

Policy information about studies involving human research participants

Population characteristics	All research participants have been diagnosed with PTEN hamartoma tumor syndrome and therefor harbor germline PTEN mutations. To address our research question, we prioritized an age- and sex-matched series of 30 PHTS individuals with autism spectrum disorder and/or developmental delay (ASD/DD; median age = 24; range = 2-58), differentiated thyroid cancer (median age = 36; range = 19-59), and those with ASD/DD in addition to a cancer diagnosis (majority having thyroid cancer; median age = 28; range = 15-51). Each phenotype group included 7 females and 3 males.
Recruitment	Inclusion criteria for enrollment into research protocol 8458-PTEN include meeting at least the relaxed International Cowden Consortium operational diagnostic criteria, meaning full diagnostic criteria minus one feature, termed Cowden-like

	syndrome; having macrocephaly plus neurodevelopmental disorders (eg, autism spectrum disorder, developmental delay, mental retardation) and/or penile freckling; or the presence of a known pathogenic germline PTEN mutation (patients referred to the PTEN Multidisciplinary Clinic at the Cleveland Clinic; Director: Charis Eng, MD, PhD).
Ethics oversight	Research protocol 8458-PTEN, approved by the Cleveland Clinic Institutional Review Board

Note that full information on the approval of the study protocol must also be provided in the manuscript.
